# Detection of SARS-CoV-2 Virus by Triplex Enhanced Nucleic Acid Detection Assay (TENADA)

**DOI:** 10.3390/ijms232315258

**Published:** 2022-12-03

**Authors:** Anna Aviñó, Carlos Cuestas-Ayllón, Manuel Gutiérrez-Capitán, Lluisa Vilaplana, Valeria Grazu, Véronique Noé, Eva Balada, Antonio Baldi, Alex J. Félix, Eva Aubets, Simonas Valiuska, Arnau Domínguez, Raimundo Gargallo, Ramon Eritja, M.-Pilar Marco, César Fernández-Sánchez, Jesús Martínez de la Fuente, Carlos J. Ciudad

**Affiliations:** 1 Institute for Advanced Chemistry of Catalonia (IQAC), Consejo Superior de Investigaciones Científicas (CSIC), 08034 Barcelona, Spain; 2 Centro de Investigación Biomédica en Red de Bioingeniería, Biomateriales y Nanomedicina (CIBER-BBN), Instituto de Salud Carlos III, 28220 Madrid, Spain; 3 Instituto de Nanociencia y Materiales de Aragón (INMA), Consejo Superior de Investigaciones Científicas (CSIC), University of Zaragoza, 50009 Zaragoza, Spain; 4 Instituto de Microelectrónica de Barcelona (IMB-CNM), Consejo Superior de Investigaciones Científicas (CSIC), Campus UAB, 08193 Cerdanyola, Spain; 5 Department of Biochemistry and Physiology, School of Pharmacy and Food Sciences, University of Barcelona (UB), 08028 Barcelona, Spain; 6 Instituto de Nanociencia y Nanotecnología (IN2UB), University of Barcelona (UB), 08028 Barcelona, Spain; 7 Department of Chemical Engineering and Analytical Chemistry, University of Barcelona (UB), 08028 Barcelona, Spain

**Keywords:** Polypurine reverse-Hoogsteen hairpin, SARS-CoV-2 RNA detection, COVID diagnosis, thermal lateral flow, electrochemical magnetoassay, fluorescent microarray

## Abstract

SARS-CoV-2, a positive-strand RNA virus has caused devastating effects. The standard method for COVID diagnosis is based on polymerase chain reaction (PCR). The method needs expensive reagents and equipment and well-trained personnel and takes a few hours to be completed. The search for faster solutions has led to the development of immunological assays based on antibodies that recognize the viral proteins that are faster and do not require any special equipment. Here, we explore an innovative analytical approach based on the sandwich oligonucleotide hybridization which can be adapted to several biosensing devices including thermal lateral flow and electrochemical devices, as well as fluorescent microarrays. Polypurine reverse-Hoogsteen hairpins (PPRHs) oligonucleotides that form high-affinity triplexes with the polypyrimidine target sequences are used for the efficient capture of the viral genome. Then, a second labeled oligonucleotide is used to detect the formation of a trimolecular complex in a similar way to antigen tests. The reached limit of detection is around 0.01 nM (a few femtomoles) without the use of any amplification steps. The triplex enhanced nucleic acid detection assay (TENADA) can be readily adapted for the detection of any pathogen requiring only the knowledge of the pathogen genome sequence.

## 1. Introduction

The COVID-19 pandemic has triggered the largest viral testing effort to monitor the spread of the infection and dictate the appropriate measures to prevent viral transmission [1]. This large testing effort has driven an extraordinary search for novel methods for the rapid detection of SARS-CoV-2 and other viral infections [2]. The reverse transcription quantitative polymerase chain reaction (RT-qPCR) method [3] provides a quantitative analysis of the viral genome, and it has been the gold standard method for the identification of infected individuals for the control of the pandemic. Although RT-qPCR can detect very small amounts of viral RNA it has been described that in the real clinical set-up it has around 50–70% sensitivity [4]. In addition, this technique requires specialized personnel and instruments with the difficulty to be performed in low-income countries with a dispersed population. Many alternative methods avoiding the higher-temperature amplification steps have been described [2] including loop-mediated isothermal amplification (LAMP, [5]), nicking endonuclease amplification reaction, and recombinase polymerase amplification (RPA, [6]). The use of CRISPR for signal amplification (SHERLOCK, [7]), has also been described [8,9]. In addition, next-generation sequencing (NGS) has provided an excellent platform for the identification of the SARS-CoV-2 genome [10,11] as well as novel SARS-CoV-2 variants [12].

The development of highly specific monoclonal antibodies against viral proteins has produced several antibody tests in simple lateral flow formats that are able to detect the presence of the virus in nasopharyngeal samples in a short time [13]. The antibody tests are very popular despite having shown less sensitivity than assays based on nucleic acids [14].

Recently we have described that DNA hairpins that recognize nucleic acid targets by triplex formation can detect Pneumocystis in bronchoalveolar lavage, nasopharyngeal aspirates, and sputum samples using surface plasmon resonance (SPR) [15] or by using nanoporous anodic alumina scaffolds filled with a fluorescent dye reporter [16]. These successful strategies make us think that triplex-forming DNA hairpins could be used for the direct detection of viral RNA, concretely the detection of single-stranded SARS-CoV-2 RNA. To this end, we analyzed the SARS-CoV-2 genome, and we found a fair number of polypyrimidine stretches suitable for designing triplex-forming hairpins using the Polypurine reverse-Hoogsteen (PPRH) strategy [17].

PPRHs are single-stranded non-modified DNA hairpins formed by two antiparallel Polypurine mirror repeat domains linked by a thymidine loop and bound by intramolecular reverse-Hoogsteen bonds. They can bind in a sequence-specific manner to polypyrimidine sequences in either ssDNA, dsDNA, or RNA by Watson–Crick bonds, thus forming an antiparallel triplex and producing strand displacement on the DNA complex [18,19]. PPRHs have been described as gene silencing tools of several genes mainly involved in cancer with the capacity to produce strand displacement [17]. Additionally, they have been incorporated as probes in biosensors for the detection of miRNA-145 [20,21], to determine the DNA methylation status of the PAX-5 gene [22], and for the diagnosis of *Pneumocystis pneumonia* [15,16].

**Figure 1:**
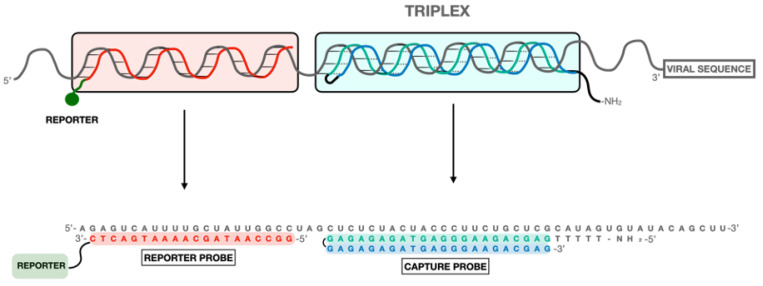
Design of the PPRH CC1 capture and reporter probes for the detection of the viral RNA.

In the case of SARS-CoV-2, we decided to use the sandwich hybridization format [23] in several biosensing devices. This strategy uses two oligonucleotides: a triplex-forming PPRH hairpin [24] acting as a capture probe and a labeled duplex-forming DNA oligonucleotide acting as a detection probe (Figure 1). The triplex-forming PPRH hairpins were designed to bind to SARS-CoV-2 polypyrimidine sequences, and the detection probes were designed to be complementary to a region near the polypyrimidine target site. In this way, the presence of the SARS-CoV-2 RNA is detected by the formation of the ternary complex in a biosensor surface. We named this method after triplex enhanced nucleic acid detection assay (TENADA).

In this communication, we describe the simple and rapid detection of viral genome by TENADA in two different biosensing devices, once the TENADA is validated by a fluorescent DNA microarray chip. The first biosensing strategy is based on a thermal lateral flow system and the second one is on a compact electrochemical biosensor platform. The results demonstrate that TENADA is highly efficient for the detection of viral RNA obtaining high sensitivity and specificity with no need for amplification making possible direct detection in a lateral flow format in less than one hour.

## 2. Results and Discussion

### 2.1. Design and Synthesis of PPRH and Reporter Probes

Our goal, based on previous experiences in diagnosing biological materials, was to develop a technique for detecting directly (without the need for PCR) and very quickly (less than 1 h), the RNA of the SARS-CoV-2 virus. The method described here takes advantage of the property of a special kind of DNA hairpin, developed in our laboratory, and named PPRHs, to capture the viral RNA forming a triplex. We searched for triplex forming sequences in the SARS-CoV-2 genome (MN938384) with more than 15 nucleotides in length, a maximum of 3 pyrimidine interruptions and a minimum of 40% of G-C content as described [24]. The output of the search was given in the form of polypurine sequences that can be found in the forward or reverse orientation (Table S1).

We selected the three longest sequences in the reverse orientation and converted the polypurine sequence (20–21 nucleotides) to the complementary polypyrimidine generating the potential targets. These targets were named as CC1 (CTCTCTACTACCCTTCTGCTC, 21 bases) located at the replicase gene position 17,111; CC2 (CCTCTTCTCGTTCCTCATCAC, 21 bases), located at the N gene position 28,806 and CC3 (TCATCTTATGTCCTTCCCTC, 20 bases) located at the spike gene position 24,690. The selected target sequences contain three purine interruptions each (underlined).

Next, we prepared the corresponding PPRHs, as described in Materials and Methods (Supplementary Material), one for each potential target (CC1PPRH, CC2PPRH, and CC3PPRH, Tables S2 and S3) in two forms: unmodified for the binding assays and extended with a pentathymidine sequence followed by the addition of an aminohexyl group at the 5′-end that will be used for the immobilization of PPRH at the surface of the biosensors.

Once the PPRHs were designed, a second oligonucleotide or reporter probe was defined with the condition that the oligonucleotide is complementary to a nearby site avoiding steric factors (Tables S2 and S3 and Figure 1).

The reporter probes were functionalized at the 3′-end with either biotin, fluorescent labels (Cy3, TAMRA), or with peroxidase protein through a reactive thiol group to provide non-radioactive labeling systems to deliver optical or electrochemical transducer signals for three different biosensors. In addition, we prepared the complementary sequence of the target polypyrimidine sites (CC1duplex, CC2duplex, CC3duplex, Table S2) to be used for comparison purposes. Finally, several synthetic DNA and RNA oligonucleotides containing the viral polypyrimidine target sequences (Table S3) were prepared for binding assays or as artificial targets for the calculation of the limit of detection of the biosensors.

### 2.2. Gel Shift Binding Assays

**Figure 2:**
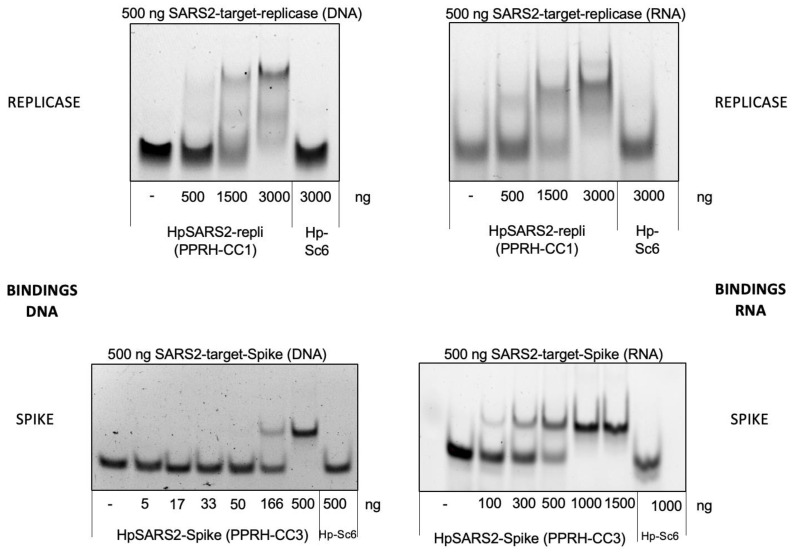
Binding of PPRHs CC1 and CC3 to DNA and RNA probes corresponding to SARS-CoV-2. Increasing amounts of PPRH-CC1 or PPRH-CC3 were incubated with 500 ng of 6-FAM-labeled ssDNA or ssRNA probes corresponding to replicase (top panel) or spike (bottom panel) sequences. Hp-Sc6 was included as a non-targeting control PPRH.

As can be seen in Figure 2, increasing amounts of the PPRHs designed against the viral sequences for the replicase or spike were able to bind to 500 ng of their corresponding targets in the SARS2 genome, either as ssDNA or ssRNA species. In both cases, a shifted band corresponding to the binding of the PPRH to its target, with lower mobility than the probe alone, can be observed. As a negative control, a PPRH with a scrambled sequence (HpSC6) was used that did not originate any shifted band in the presence of the specific probes.

Gel shift assays on polyacrylamide gels allowed the measurement of the dissociation constant of the triplex-forming probes and the duplex-forming probes with their target sequence labeled with fluorescein. The dissociation constants (*K*_d_) have been determined for the formation of the triplex and the duplex, which are detailed in Figure S1 and Table S4. A control oligonucleotide (CC1-control) was added. This oligonucleotide can only form a duplex because the reverse Hoogsteen strand is scrambled. PPRH-CC1 and PPRH-CC3 present the highest affinity for their targets with dissociation constants of around 3.8 × 10^−7^ M. PPRH-CC2 presented the lower affinity PPRH (*K*_d_ 6.04 × 10^−7^ M). As PPRH-CC1 and PPRH-CC2 have the same length (21 nt) and GC content (47.6%, Table S1) we believe that the reason for the lower affinity may be due to the relative position of the three interruptions (in CC2 the three interruptions are near the ends of the hairpins and this may debilitate the formation of the hairpin). However, all PPRHs have a higher affinity than the corresponding duplex-forming oligonucleotides (duplex-CC1, duplex-CC2, duplex-CC3, PPRH-CC1-control, *K*_d_ between 4.79 and 10.5 × 10^−7^ M). These data demonstrated a greater affinity of the triplex-forming probes for their target. (Figure S1 and Table S4).

### 2.3. CC Pair Validation with a Fluorescent DNA Microarray Chip

**Figure 3:**
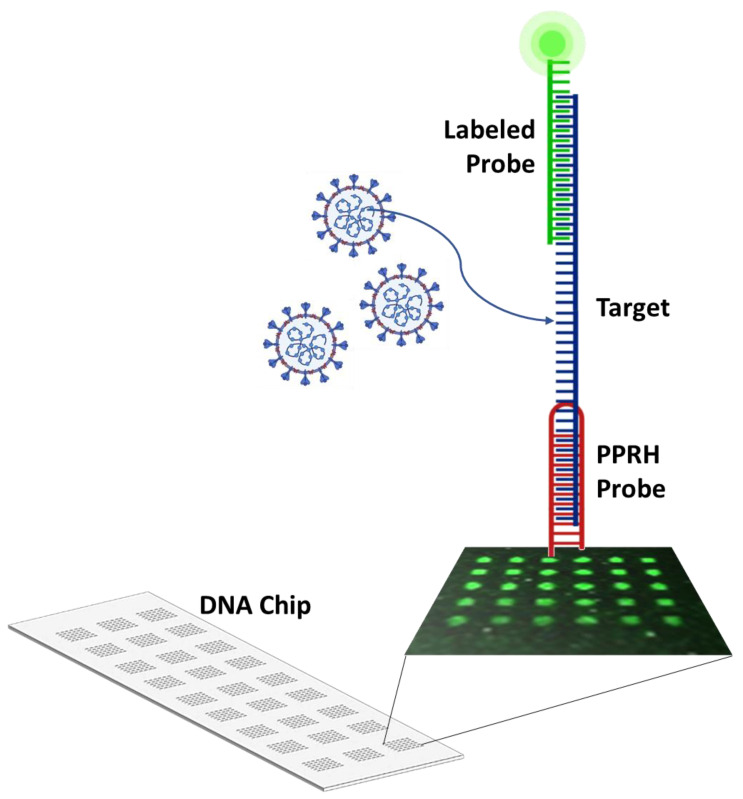
Scheme of the microarray system developed to validate the CC pairs designed for detecting SARS-CoV-2 viral RNA. It comprises a glass surface onto which DNA capture probes (PPRH or duplex) are chemically bounded. These capture probes printed on the glass slide hybridize selectively with the corresponding synthetic target and this binding is reported through the addition of a second probe, which allows the detection since it is coupled to a fluorophore. Thus, the principle of the system is based on the complementarity base pairing between the probes and the corresponding target nucleotide sequence.

Since microarray analysis is a well-established and reliable methodology to study hybridization events between specific probes and the complementary targets, it has been used to validate the design and performance of the different probe pairs (CC capture and detection probes) in both formats: PPRH and duplex. With this aim, an array has been developed printing on a solid surface the different capture probes that hybridize selectively with the corresponding synthetic target sequences. This hybridization process is reported to add specific labeled detection probes. More precisely, the capture probes (first oligonucleotide) have been chemically bound to a glass slide through the addition of a capture tag, which in this case is an amino group that reacts with the isothiocyanate groups of the previously biofunctionalized surface. These printed oligonucleotides bind to the corresponding target nucleotide sequences by complementarity base pairing events which are proved by adding specific detection probes (second oligonucleotide) labeled with a fluorophore (TAMRA or Cy3) (Figure 3). Finally, data acquisition is achieved by exciting the fluorophore with a laser beam of 532 nm. This readout signal is then scanned with a microarray scanner to visualize and quantify spot fluorescence.

To find out the optimal microarray working conditions, single oligonucleotide pair standard curves were performed printing six different concentrations (from 500 nM to 0 with a dilution factor of 2) of the capture probe (in PPRH and duplex format). Subsequently, eight serial dilutions of the corresponding target (ranging from 500 nM to 0 with a dilution factor of 5 and prepared in hybridization buffer) were added and let for hybridization during 5 min at RT. Finally, the detection probe was incorporated and incubated for 10 min at RT assaying two different concentrations of each one of them (500 and 250 nM also prepared in hybridization buffer). With the data obtained from these experiments, the analytical parameters of each CC pair microarray assay were determined. Afterward, matrix effect studies to assess the performance of the developed chip when diluting each target with different universal transport media (UTM, i.e., the buffer used to collect the swabs) were also carried out. This study was needed since this sort of sample is usually collected in tubes that contain a UTM which, depending on the brand, can slightly vary its composition and either include the inactivation agent (usually guanidine isothiocyanate) or not. In our case, the matrix effect was studied by testing three different universal transport media (UTM) named buffers 1, 2, and 3. In all cases, good analytical features were obtained and even a slight improvement was observed when compared with the results obtained with the hybridization buffer prepared in the lab (see these data in Table S5).

**Figure 4:**
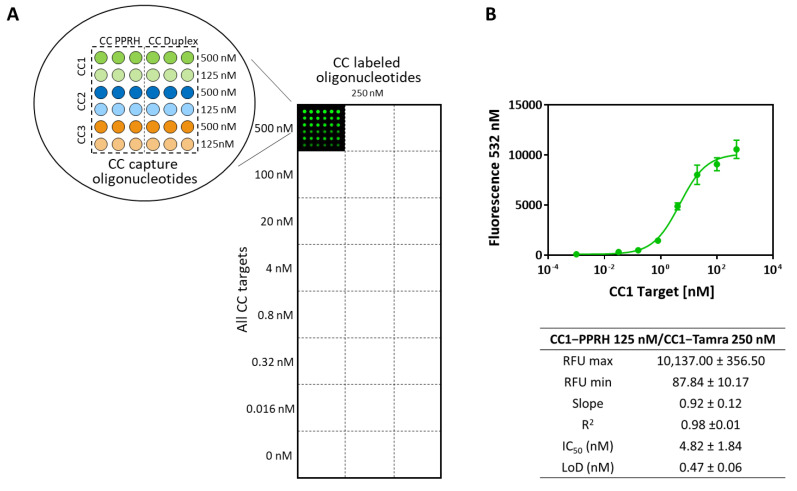
Microarray system designed to test simultaneously (multiplex) all three CC oligonucleotide pairs assayed in both formats (PPRH and duplex clamp). (**A**): Scheme of the chip organization and the concentrations used to build standard curves for each CC pair are shown together with a fluorescence image of a microarray well. (**B**): Calibration curve obtained for the CC1 pair and the corresponding analytical parameters of this sigmoidal curve obtained in a multiplex microarray assay (RFU: relative fluorescence units) are also plotted. All standard curves obtained were designed to record at least 3 spot replicates for each concentration value.

Moreover, cross-reactivity studies were carried out to identify possible nonspecific hybridization events between oligonucleotides from different sets or among an oligonucleotide and a non-specific target. These experiments showed that neither targets nor CC oligonucleotides caused cross-hybridization artifacts, demonstrating that all hybridization processes that take place are specific (see Figure 4). These results also confirm the feasibility of developing a multiplex assay with the CC pairs studied. Finally, aiming at reproducing the conditions that will occur when testing real clinical samples, the synthetic CC1 DNA target used to develop the assay was replaced by the equivalent one but in an RNA format. The standard curve built in this case did not show significant differences with the ones obtained with DNA synthetic target confirming that the hybridization DNA: RNA is also optimal.

### 2.4. First Biosensor Device Thermal Lateral Flow System

**Figure 5:**
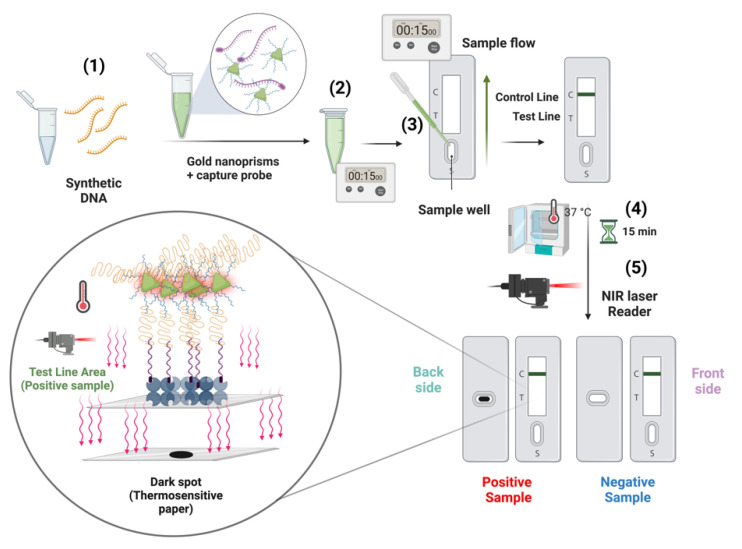
Scheme of the thermal lateral flow system and the analytical procedure. 1—Sample addition to a solution containing gold nanoprisms and the capture oligonucleotide labeled with biotin; 2—preincubation; 3—preincubated sample is added to the TLF strip, leaving the samples flow 15 min; 4—sample drying at 37 °C for 15 min; 5—test developing by NIR laser irradiation.

**Figure 6:**
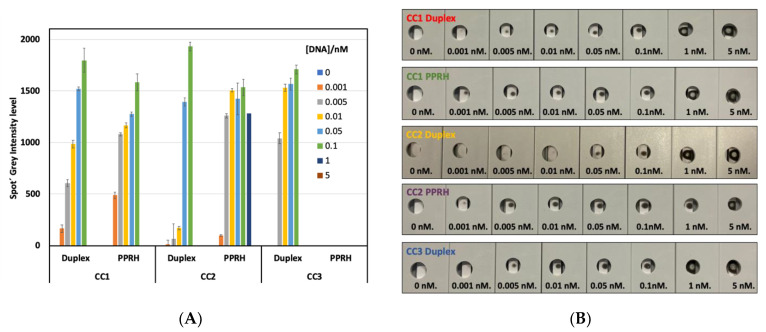
Results obtained with the thermal lateral flow system. (**A**): Profile of ipeak^®^ (IUL S.A. commercial lateral flow reader) quantification of spiked samples with different concentrations of targets (from 0 to 5 nM) in UTM using capture sequences duplex-CC1, CC2, and CC3 duplex and PPRH-CC1 and CC2. Results with PPRH-CC3 sequence are not shown in this graph because NPRs aggregation was observed during the testing assay. Grey level intensity measurements were obtained from the LF reader to determine the value of LOD of the thermal lateral flow system. Standard deviation of three replicates carried out consecutively is drawn as error bars. (**B**): Image obtained from the back part of the LF strips (thermosensitive material) after the laser irradiation using a 1064 nm laser. The strips were loaded with spiked samples in UTM from Biocomma with different concentrations of DNA, from 0–5 nM, to determine the visual LOD of the system.

The first technology developed to detect SARS-CoV-2 RNA is based on a commonly used lateral flow assay (Figure 5). Lateral flow technologies are based on the detection of different kinds of biomolecules by using a nitrocellulose membrane platform where a test line and a control line have been previously deposited. The test line is composed of a protein solution (3 mg/mL) which recognize specifically the biotinylated capture probes after the interaction with the desired target. The control line is formed by a solution of a modified protein (2 mg/mL) with a particular oligonucleotide that recognizes the detection probe (first oligonucleotide) introduced in the gold nanoprisms (NPr). The detection probes or first oligonucleotides have been, previously to the biofunctionalization, modified by the introduction of a terminal amino group allowing the attachment onto the carboxylic derived gold nanoprisms. The concentration of detection oligonucleotide used in the biofunctionalization has been optimized at 1.36 pmol/µL. These probes hybridize selectively with the corresponding target and this binding was completed through the addition of a second capture probe. This second capture probe allowed the detection since it was labeled with biotin which reacts with the streptavidin introduced at the test line. The corresponding target was tested at eight different concentrations (ranging from 5 nM to 0). The LoD has been calculated after the membrane development by using a 1064 nm NIR laser; see Figure 6a. Visual detection has been performed in the back part of the strip by using a thermosensitive paper which changes from white to black when the local temperature increases as a consequence of plasmonic nanoparticles (gold nanoprisms, NPr) laser irradiation, see Figure 6b. No results are shown for PPRH-CC3 because nanoparticle aggregation was observed once the particles were introduced in the LF strip. The highest sensitivity was achieved with PPRH-CC1 and PPRH-CC2 with a limit of detection between 0.01 and 0.005 nM. The control duplex-CC1 and CC2 gave a limit of detection between 0.1 and 0.05 nM (one order of magnitude less sensitive). The matrix effect was studied testing one universal transport media (UTM) from Biocomma Limited (Guangdong, China) which has been selected due to the better compatibility with the TLF system. Similar results were obtained, even important improvements were observed when the results were compared with the corresponding in buffer media, as better LOD was obtained in all cases.

### 2.5. Second Biosensor Device Compact Electrochemical Biosensor Platform

**Figure 7:**
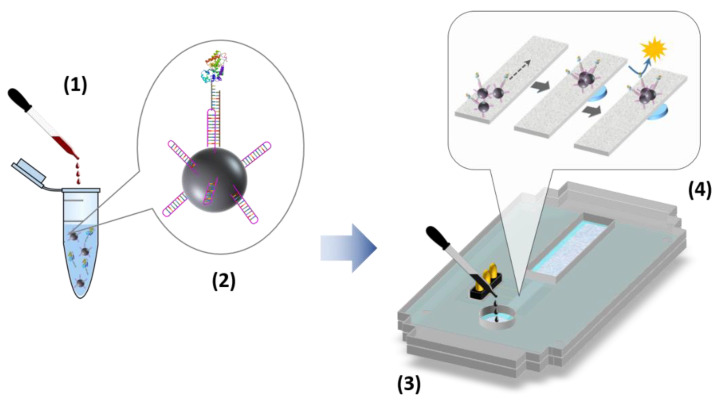
Scheme of the electrochemical biosensor platform and the analytical procedure: 1—sample addition to a solution containing functionalized MNPs and the detection oligonucleotide labeled with HRP enzyme; 2—hybridization assay on the MNP surface; 3—pretreated sample added to the electrochemical device; 4—steps taken place in the electrochemical device, including MNP flow, trapping and concentration, and electrochemical detection.

**Figure 8:**
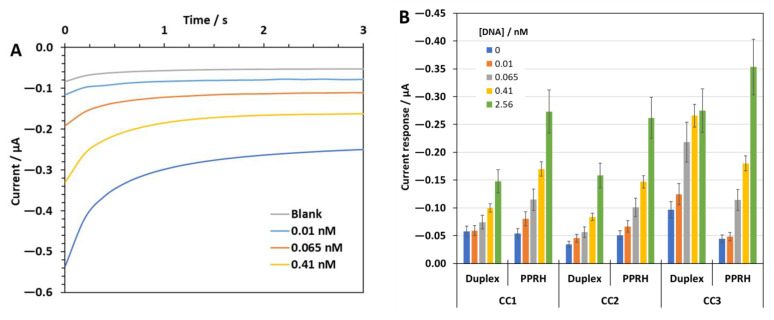

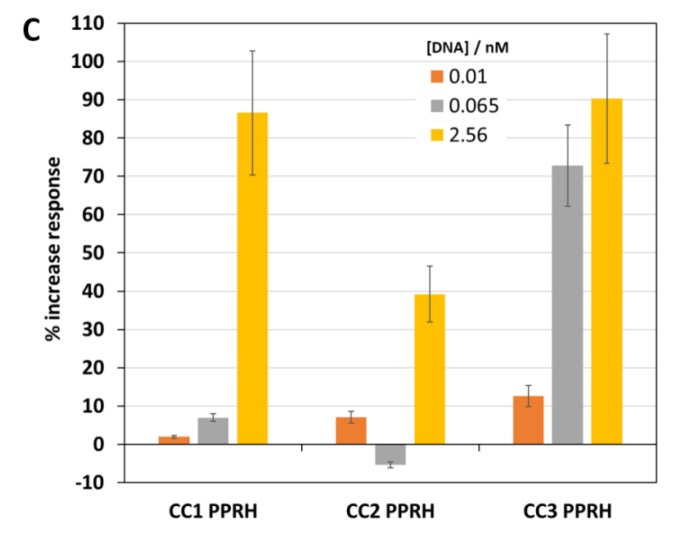
(**A**): Current response profiles to different concentrations of the target DNA CC1 sequence in hybridization buffer using the MNPs modified with PPRH—CC1. (**B**): Analytical signal values recorded with the three different capture probes, CC1, CC2, and CC3, in a duplex and PPRH format for different concentrations of the corresponding oligonucleotide target DNA sequences in hybridization buffer. (**C**): Increase in response relative to the blank response (in %) recorded in the UTM from Biocomma with the PPRH—CC1, CC2, and CC3, respectively, for three of the tested oligonucleotide concentrations. Standard deviation of three replicates carried out consecutively is drawn as error bars.

Next, a compact fluidic electrochemical biosensor platform was developed (Figure 7) chronoamperometric responses to four different target sequence concentrations plus the blank signal in the hybridization buffer were recorded. As an example, Figure 8A depicts the signals obtained using the MNPs modified with PPRH-CC1. As expected, the cathodic currents increase with the target concentration, 0.01 nM (1 fmol) was the lowest concentration providing a signal that differed from the blank.

Figure 8B shows a bar graph bar comparing the analytical signals recorded with the six different capture sequences tested. In general terms, the PPRH—sequences present higher absolute current values and better sensitivities than the duplex ones. Then, the PPRH sequences were used to evaluate the electrochemical sensor performance in the UTM from Biocomma, as an approach to simulate the real conditions of analysis. Figure 8C shows bar graphs comparing the increase in the biosensor response with respect to the blank signal recorded in the UTM (in percentage) for PPRH-CC1, CC2, and CC3. Although a general decrease in the absolute current values was observed due to some possible matrix effects, the minimum concentration measured was also 0.01 nM in most cases. However, some important differences were observed among the different PPRH, CC2 showed a poor response. By contrast, the PPRH-CC1 appeared to keep a quite good response in the UTM with good proportionality with the target concentration and a wider linear range of response. Therefore, MNPs modified with PPRH-CC1 were used for the detection of SARS-CoV-2 RNA in clinically relevant samples.

### 2.6. Detection of SARS-CoV-2 RNA in Clinically Relevant Samples

**Figure 9:**
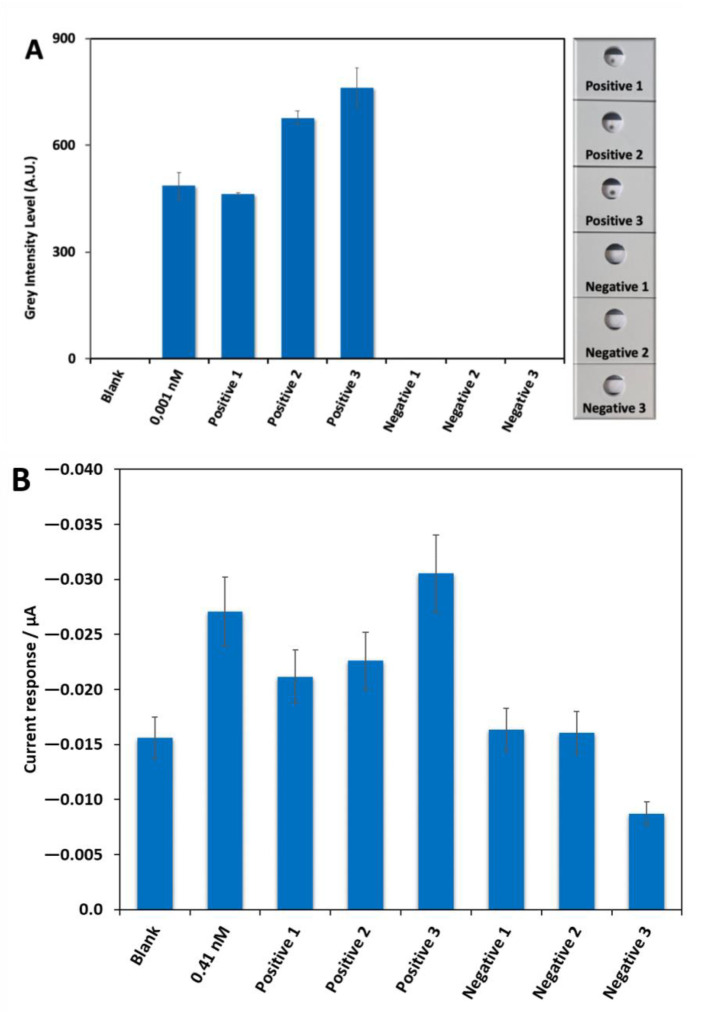
Testing of six real samples provided by the Biobank of the Aragon Health System (3 negatives and 3 positives by PCR: sample #1, Ct 35; sample #2, Ct 19 and sample #3, Ct 30) in UTM from Biocomma with the PPRH-CC1 as capture sequence using the two developed biosensor devices. (**A**): Profile of ipeak^®^ (IUL S.A. commercial lateral flow reader) quantification (grey intensity level measurements, right side) of the results obtained with the TLF together with the signal obtained for the blank and control of 0.001 nM of target DNA CC1 sequence and the image obtained from the back part of the LF strips (thermosensitive material, left side) after the laser irradiation using a 1064 nm laser used. This image was used to measure the grey level intensity to develop the results of the thermal lateral flow assay. (**B**) Analytical signal values obtained by the electrochemical biosensor platform, together with the signal obtained for the blank and control of 0.41 nM of target DNA CC1 sequence. Standard deviation of three replicates carried out consecutively is drawn as error bars.

To demonstrate the applicability of the two developed biosensor systems, a set of six clinical samples provided by the Biobank of the Aragon Health System (three positives and three negatives by PCR) were analyzed simultaneously. The samples were collected in the UTM from Biocomma. In the case of the TLF system evident dark spots were obtained in the back part of the LF strips, after being irradiated using NIR laser, for the three positive samples and no signals were obtained for the negatives ones (Figure 9A right side). A quantification profile was also performed using ipeak^®^ (IUL S.A.) commercial lateral flow reader (Figure 9A left side) showing that the signal obtained for the positive 3 was higher than the signal obtained for positive 2 and this one higher than the obtained for positive 1. The graph also exposed that the signal obtained for the positive 1 was similar to the signal of the control of 0.001 nM and the values obtained for negative samples 1, 2, and 3 were very similar to the blank. In the electrochemical system, a clear increase in the analytical signal was observed for the three positive samples with respect to the negative ones (Figure 9B). In fact, the values for negatives 1 and 2 samples were very similar to the blank. The lower signal recorded for negative 3 compared with that of the blank is likely to be due to the sample matrix, collected by swabbing the patient’s nasopharyngeal cavity. The biological material that is dissolved in the UTM could be very complex, and a matrix effect on the sensor response may take place, resulting in a signal that is lower than that of the blank signal recorded in a fresh UTM. Regarding the positives, the signal for the positive 3 samples was higher than the control of 0.41 nM. The Cts for the three positive samples analyzed were 35 (sample #1), 19 (sample #2), and 30 (sample #3). Obviously, sample #2 with a Ct value of 19 was clearly positive, as it also was using our system. However, interestingly enough, the other two samples with much higher Ct values, and very especially sample #1 with Ct = 35, in the borderline between positive and negative in the PCR, gave a positive signal using the TENADA method. It should also be noted that the trend regarding the absolute signal values recorded for the three positive samples is the same for the two developed systems.

The two strategies described here especially using PPRHs as capture probes could be used as point-of-care screening for detecting SARS-CoV-2 and other viruses without the need of a pre-amplification step, as an alternative to other methods such as CRISPR-Cas13a [7,8,9], RPA [6], LAMP [5] and LAMP-Seq [25] developed recently. The limit of detection of TENADA was around 0.01 nM (a few femtomoles). Detection of the presence of the RNA of the SARS-CoV-2 virus in nasopharyngeal samples agreed with the results obtained by PCR. Alternative methods [5,6,7,8,9,25] may be more sensitive but the time required for the analysis in TENADA is less than 1 h, shorter than most of these alternative methods [5,6,7,8,9,25]. The detection limit, the analysis time, and the requirement of simple instrumentation are strong arguments for further development. During the two years of the pandemic, many mutations have been described. We have checked if any of the known mutations are in the selected target sequences and, until now, these mutations did not affect the target sequences, indicating that the selected polypyrimidine targets are well preserved.

## 3. Materials and Methods

### 3.1. Design of PPRH

The polypyrimidine sequences of SARS-CoV-2 genome were searched using the triplex-forming oligonucleotide Target Sequence Search software (Version V.1) from University of Texas, Austin, TX, USA (http://utw10685.utweb.utexas.edu/tfo/, accessed on 1 November 2022). The default parameters were set as (1) a minimum of 15 nucleotides in length; (2) a minimum of 40%GC content; and (3) a maximum allowable of 3 pyrimidine interruptions. In these conditions, we selected the longest three polypyrimidine sequences (20–21 nucleotides) that were checked by BLAST +2.13.0 software to be not present in another virus or human genome (https://blast.ncbi.nlm.nih.gov/Blast.cgi?PAGE_TYPE=BlastSearch, accessed on 1 November 2022). The PPRH capture oligonucleotides were formed by the selected polypurine sequence followed by a 4-thymidine loop and a mirrored sequence to form a hairpin by intramolecular reverse-Hoogsteen bonds (see Tables S1 and S2). We designed three different hairpin oligonucleotides CC1, CC2, and CC3. For comparative purposes, purine duplex capture oligonucleotides were also prepared. The second oligonucleotide to perform the sandwich hybridization strategy was complementary to a 20-nucleotide sequence located near, usually 3 nucleotides 5′, to the polypyrimidine target sequence (Figure 1).

### 3.2. Synthesis of Oligonucleotides

Oligonucleotide sequences were obtained either from commercial sources (Merck/Sigma, Haverhill, UK) or synthesized on an automatic Applied Biosystems 3400 DNA synthesizer on a 0.2 µmol (LV200) scale using commercially available chemicals. Oligonucleotides were prepared using standard phosphoramidite solid-phase protocols. The introduction of amino groups at the 5′-position was performed by using *N*-trifluoroacetyl-6-aminohexyl 2-cyanoethylphosphoramidites. The introduction of the reporter groups such as biotin (biotin-TEG) or fluoresceine (FAM) or Cy3 was performed using the appropriate phosphoramidite (if added 5′-end) or functionalized controlled-pore glass (CPG) (if added at the 3′-end). After the assembly of the sequences, oligonucleotide-supports were treated with 32% aqueous ammonia at 55 °C for 16 h. Ammonia solutions were concentrated to dryness and desalted by Sephadex G-25. Alternatively, the products were purified by cartridge oligonucleotide purification (COP) columns.

### 3.3. Gel-Binding Assays

Binding experiments were carried out by incubating the 6-FAM-labeled ss DNA or RNA probes corresponding to the SARS-CoV-2 targets with the corresponding PPRHs in a buffer containing 10 mM MgCl_2_, 100 mM NaCl, and 50 mM HEPES (pH 7.2), supplemented with 5% glycerol. Binding reactions (20 μL) were incubated 30 min at 37 °C. A scrambled PPRH (HpSC6: AAGGAAGGAAGGAAGGAAGGAAGGTTTTTGGAAGGAAGGAAGGAAGGAAGGAA) was used as negative control. Electrophoresis was performed on nondenaturing 8% polyacrylamide gels containing 10 mM MgCl_2_, 5% glycerol, and 50 mM HEPES (pH 7.2). Gels were run at a fixed voltage of 190 V (4 °C) using a running buffer containing 10 mM MgCl_2_ and 50 mM HEPES (pH 7.2). Finally, gels were visualized using the Gel Doc™ EZ with the Image Lab Software, Version 6.0 (Bio-Rad, Barcelona, Spain). All reagents were obtained from Sigma-Aldrich (St. Louis, MO, USA).

### 3.4. Fluorescent DNA Microarray Chip

This protocol describes the chemical derivatization of the microarray slides, the spotting of the oligonucleotides, the hybridization reaction, and the fluorescence signal detection.

Slide derivatization: plain glass slides were first cleaned by immersing them in Piranha solution (H_2_SO_4_:H_2_O_2_ 70:30 *v*/*v*) for 30 min. Subsequently, they were rinsed with Ultrapure water, activated with 10% NaOH for 2 h, and then rinsed again with ultrapure water and with ethanol 60% solution. After this cleaning step, they were dried with N_2_. Next, the chemical functionalization of the slides was achieved using a silane reagent. The protocol followed takes place in two steps: first, the aminopropyltrimethoxysilane (APTMS) reacts with the hydroxyl groups of the glass surface for 3 h at RT; and after, the amino groups of the silane react with the isocyanate groups of 1,4-phenylene diisothiocyanate during 2 h at RT in 10% pyridine. After this treatment, slides were serially washed with a 60% ethanol solution, methanol, and acetone, dried with N_2_, and stored in the desiccator until use.

DNA microarray chip manufacture: the first oligonucleotide solutions were prepared in printing buffer (150 nM sodium phosphate, 0.01% SDS) and subsequently filtered with 0.45 µM PVDF syringe filters (Millipore). Afterward, they were spotted in defined positions of the derivatized glass slides. This printing step was carried out using a sciFLEXARRAYER S3 (Scienion), under controlled temperature (20 °C) and humidity (65%). To perform the printing of each spot 5 drops were dispensed with a PDC70 piezo dispensing capillary (350 pL/drop). At the end of the whole printing process, slides were kept for 30 min inside the microarrayer chamber. The biofunctionalized slides were then stored at 4 °C until use. Up to 24 microarray chips could be printed in a single slide. Microarray chips for multiplex analysis consisted of a matrix of 6 × 6 spots (3 different sets of oligonucleotides (first and second oligonucleotides) including the PPRH and duplex clamp format and 3 replicates of each). This scheme could be easily modified to allow the analysis of a higher number of samples.

Hybridization assay: the slides were placed on an Arrayit holder provided with a silicon gasket defining 8 × 3 wells on each slide. Before starting the assay, the slides were washed (200 µL/well) three times with PBST (0.01 M phosphate buffer in a 0.8% saline solution at pH 7.5 plus 0.005% Tween 20). Target standard solutions were prepared in Hybridization buffer (10 mM Tris, 1 mM EDTA, 1 M NaCl, adjusted at pH 7.2) or in the corresponding universal transport media used to collect the samples to be analyzed (7 concentration points plus zero: 0.032 nM–500 nM). Then, samples or standard solutions were added (50 µL/well) and incubated at RT for 5 min. The next hybridization step included the addition of the second Cy3 labeled oligonucleotide (50 µL/well). After 15 min at RT, the slides were washed again (200 µL/well) three times with PBST and once with ultrapure water and finally dried with N_2_.

Fluorescence signal detection: microarray fluorescence measurements were recorded on an InnoScan 710 (Innopsys) with an optical filter of 532 nm. The spots were measured by subtracting the mean of the corresponding fluorophore background intensity to the mean of the fluorophore foreground intensity using Mapix (Innopsys) software. The standard curves were analyzed with a four-parameter logistic equation using the software GraphPad Prism. Thus, the equation follows the formula: [(A − B)/1 − (x/C)D] + B where A is the maximal fluorescence, B the minimum, C the concentration producing 50% of the difference between A and B (IC_50_ value), and D the slope at the inflection point of the sigmoid curve. The limit of detection (LoD) was defined as the concentration producing 10% of the maximal fluorescence (IC_10_ value).

### 3.5. Thermal Lateral Flow System Using PPRH as Biosensors Linked to Gold Nanoprisms

All reagents used in this biosensor’s development were of high purity, analytical grade or equivalent and were purchased from Sigma-Aldrich (Madrid, Spain).

#### 3.5.1. Gold Nanoprisms (NPrs) Synthesis

Gold NPrs with a plasmon band between 1000 and 1200 nm have been prepared following a methodology previously reported by Pelaz et al. 2012 [26] prior to use, all glassware was washed with aqua regia and rinsed thoroughly with Milli-Q water.

To perform the synthesis, 140 mL of 0.5 mM Na_2_S_2_O_3_ (11 mg, 70 µmol) aqueous solution (Milli-Q water) containing 10 μL of 0.1 M KI (0.16 mg, 1 µmol) was added slowly to 200 mL of aqueous HAuCl_4_•H_2_O_2_ mM (136 mg, 400 µmol) in a slow but continuous way during 30 s. After 4 min, another 140 mL of Na_2_S_2_O_3_ 0.5 mM (11 mg, 70 µmol) containing 10 µL of KI 0.1 M (0.16 mg, 1 µmol) was added. After another undisturbed 4 min, 60 mL of Na_2_S_2_O_3_ 0.5 mM (4.7 mg, 30 µmol) were slowly added to the solution and the resulting mixture was left reacting for an hour at room temperature avoiding the light covering the mixture with aluminum foil.

UV–VIS (Cary-50, Variant) spectra revealed a strong absorbance peak at 1000 nm corresponding to gold nanoprisms as well as a minor absorption band at 536 nm corresponding with the by-product gold pseudospherical (polyhedral) nanoparticles. The concentration of NPrs was calculated using their LSPR peak absorbance at 1050 nm and applying a conversion factor (ε) 29 mL mg^−1^ cm^−1^. Note that ε was obtained from combined UV–VIS spectroscopy/ ICP analyses.

Gold NPrs were stabilized using heterobifunctional HS-PEG-COOH (Mercapto-ω-carboxy PEG MW. 5000 Dalton) by conjugation to the gold surface by the thiols (SH-) functional groups. For this purpose, a solution of HS-PEG5000-COOH (aq.) with a ratio PEG:Au NPrs of 2:1 (in mg) was added to the nanoprisms solution. HS-PEG5000-COOH was diluted in 1-mL Milli-Q and a determined volume of 10 mg/mL stock solution of NaBH_4_ was then added to reach a 1:1 molar ratio of PEG: NaBH_4_. After that, the pH was raised to 12 with the addition of aqueous 2 M NaOH under mild mixing and the solution was sonicated for 30 min at 60 °C to complete the reaction with HS-PEG5000-COOH.

The resultant mix of gold nanoprisms and spheres was centrifuged at 5500 rcf for 15 min at room temperature to remove unreacted reagents and unwanted by-products. While the supernatant was discarded, the precipitate was resuspended in the same volume of water and two further washing steps were performed with Milli-Q water using the same conditions.

The aqueous dispersion of PEG derivatized gold nanoprisms and gold nanospheres (2 mL, 1.5 mg/mL) were loaded (mixed with loading buffer, i.e., TBE 0.5×, 5% glycerol) in wells within an agarose gel (2.5%) immersed in an electrophoresis cuvette filled with TBE 0.5×. Electrophoresis separation was run at 120 V for 40 min. The higher electrophoretic mobility and lower hydrodynamic diameter of nanospheres compared to nanoprisms, allows the nanospheres to enter in the gel and the nanoprisms stayed in the wells. The green nanoprisms solution was recovered from the wells carefully with a micropipette. The resultant dispersion of gold nanoprisms in TBE 0.5× was centrifuged and washed with Milli-Q water (3 times in total) at 5500 rcf for 15 min at room temperature to remove the TBE buffer.

#### 3.5.2. Nanoprisms Biofunctionalization

PEG derivatized gold nanoprisms were functionalized using amine-modified oligonucleotides able to recognize specific regions of COVID-19 viral RNA (PPRHs-CC1, CC2 or CC3 or duplex-CC1, CC2, or CC3). Briefly, 0.25 mg of PEG-derivatized gold nanoprisms were incubated with EDC (*N*-(3-dimethylaminopropyl)-*N*’-ethylcarbodiimide hydrochloride) 3 mM and Sulfo-NHS (*N*-Hydroxysulfosuccinimide sodium salt) 7 mM in 0.5 mL of filtered 10 mM MES (2-morpholinoethanesulfonic acid monohydrate) buffer pH 6 for 30 min at 37 °C; after that, the activated gold nanoprisms were centrifuged 9 min at 6500 rpm. After removal of the supernatant, the nanoprisms were incubated for 1.5 h at 37 °C with 0.68 nmol of amino-modified oligonucleotides (PPRHs–CC1, CC2, or CC3 or duplex-CC1, CC2, or CC3), 0.5 mL in filtered 50 mM MES buffer pH 6). Then, 0.5 mL of alpha-methoxy-omega-amino polyethylene glycol (MeO-PEG-NH_2_, 750 Da) in filtered 50 mM MES buffer pH 6 were added to the nanoprisms solution for 2 h at 37 °C added to derivatize the remaining activated carboxylic groups. Finally, biofunctionalized nanoprisms were washed out of ligand excess by centrifugation; nanoprisms were centrifuged three times for 9 min at 5500 rpm, and then pellets were resuspended in filtered 10 mM Hepes (4-(2-Hydroxyethyl)piperazine-1-ethanesulfonic acid) buffer pH 7.2 0.1% Tween 20, 0.1% BSA.

#### 3.5.3. Capture Molecules (Test and Control Lines) Preparation

Test line’s capture molecule was prepared by dissolving streptavidin (streptavidin from *Streptomyces avidinii*) at 1 mg/mL in Milli-Q water.

Control line´s capture molecule was prepared by incubation of carboxylic acid-modified oligonucleotides able to recognize the nanoprisms biofunctionalized with first oligonucleotide (PPRHs-CC1, CC2, or CC3-amino or duplex-CC1, CC2 or CC3-amino) with EDC (*N*-(3-dimethylaminopropyl)-*N*’-ethylcarbodiimide hydrochloride) 1.5 mM and Sulfo-NHS (*N*-hydroxysulfosuccinimide sodium salt) 3 mM in 0.05 mL of filtered 10 mM MES (2-Morpholinoethanesulfonic acid monohydrate) buffer pH 6 for 30 min at 37 °C. After that, a solution of BSA (bovine serum albumin) 2 mg/mL (0.05 mL) in filtered 10 mM MES buffer pH 6 was added for 2 h at 37 °C. Then, the bioconjugate was centrifuged for 14 min at 14,000 rpm using a 3 K Amicon Ultra tube to concentrate it. The final concentration is determined by UV spectrophotometer at 280 nm.

#### 3.5.4. Preparation of Lateral Flow Test Strips

The TLF (thermal lateral flow) system was composed of 3 parts, held together in polyvinyl chloride (PVC) backing card sheet with a geometry of 80 mm × 300 mm: a glass fiber conjugate Pad (8964, Ahlstrom-Munksjö), which can be considered also as a sample pad), a nitrocellulose membrane (FF80HP, GE Healthcare Life Sciences, Little Chalfont, UK), and a cellulose wicking pad (222, Ahlstrom-Munksjö) which facilitates the liquid flow (Figure 5).

To prepare the capture and control regions, a solution of both capture and control molecules, at 1 and 3 mg/mL, respectively, in MilliQ water and PBS 10 mM (Pan Biotech, GmbH) were applied to the nitrocellulose membrane using a KinBio XYZ Platform Dispenser HM3030/HM3035 (Kinbio Tech.Co., Ltd. Shanghai, China). The nitrocellulose membrane was incubated after the test and control lines dispensing at 37 °C for 1 h in an incubator. After the test and control molecules solutions dried, the LFA strip parts were mounted onto the adhesive PVC backing card, and all the system was covered by a plastic film, which ensured correct contact between parts and avoided evaporation process during the assay. The final assembly was cut into 0.4 mm wide strips, and they were stored in a dry place.

#### 3.5.5. Thermal Lateral Flow Assay (TLFA) Methodology

An amount of 25 μL of a spiked sample solutions containing increasing concentrations of the target DNA sequence complementary to the capture probes (0, 0.001, 0.005, 0.01, 0.05, 0.1, 1, and 5 nM) in Universal Transport Media (Biocomma Ltd., Shenzhen, China) were tested by mixing with 10 μg of nanoprisms functionalized with amino modified first (PPRH-CC1, CC2, or CC3-amino or duplex-CC1, CC2 or CC3) oligonucleotides, 0.0638 nmol (26.6 μL) of a biotinylated second oligonucleotide and 23.4 μL of running buffer. This mix was preincubated during 15 min at room temperature. Then, 0.1 mL of mix was loaded into the strip’ cassette. The chromatography was performed for 15 min and after that the strip was dried at 37 °C for 15 min. The development of the stripes was performed by using a NIR laser (1064 nm, 1200 mW, 1 min). Positive samples will provide a dark brown spot after laser irradiation on the nitrocellulose strip when positive samples with high concentration of synthetic DNA were analyzed) and/or in the thermosensitive paper on the back side of the nitrocellulose which were included to increase the sensitivity of the sensor. A scheme of the process can be seen in Figure 5.

### 3.6. Electrochemical Biosensor

All reagents used in this approach were of high purity, analytical grade, or equivalent and were purchased from Sigma-Aldrich (Spain), unless stated otherwise. An amount of 200 nm diameter carboxylated magnetic nanoparticles (MNPs, fluidMAG-ARA, Chemicell GmbH, Germany), 1-ethyl-3-(3-dimethylaminopropyl)carbodiimide hydrochloride (EDC), 0.1 M 2-(*N*-morpholino)ethanesulfonate (MES) buffer pH 5.0, phosphate-buffered saline (PBS) solution pH 7.4, 50 mM tris(hydroxymethyl)aminomethane (TRIS) buffer pH 7.2, 0.1 M citrate/acetate buffer pH 5.5, ferrocene-methanol and H_2_O_2_ were utilized. The oligonucleotides used were: the capture sequences (PPRH-CC1, CC2, and CC3; duplex-CC1, CC2, and CC3), the target sequences CC1, CC2, and CC3, and the thiol-reported sequences (RP-CC1, CC2, and CC3), respectively. These three thiol-reported sequences were labeled with the horseradish peroxidase (HRP) enzyme using an HRP-oligo conjugation kit (thiol oligo) from CellMosaic, Inc. (https://www.cellmosaic.com/content/Manual/DCM53402_HRP_Oligo_Kit_VB.pdf; accesed on 23 April 2021, Woburn, MA, USA).

The compact fluidic electrochemical biosensor platform comprises the following main components. A scheme can be seen in Figure 7.

1. A reusable electrochemical cell of two gold thin-film electrodes fabricated by a standard photolithographic/lift-off process on 4-inch silicon wafers at the IMB-CNM Clean Room facilities [27]. Additionally, 8 × 8.3 mm^2^ silicon chips, each one including a 1 × 1 mm^2^ working electrode and a 1.5 × 1 mm^2^ counter/reference electrode were manufactured.
2. A disposable fluidic channel made of Whatman^®^ cellulose chromatography paper, Grade 1, cut using a custom-made die cutter (Tecnocut, Barcelona, Spain) sandwiched between two polyvinyl layers patterned using a blade plotter (CAMM-1 Servo Cutter, Roland DG, Barcelona, Spain) to expose the fluidic channels in the sample addition and detection areas.
3. A poly(methyl methacrylate) cartridge to integrate and align the cell and the fluidic channel, machined using a CO_2_-laser printer (Epilog Mini 24, Epilog Laser, Golden, CO, USA). The bottom part of the cartridge included an Nd magnet to trap MNPs inside the platform, as explained below.

The performance of the biosensor comprises the use of MNPs functionalized with the required capture sequences. The functionalization process was carried out as follows. An amount of 5 mg of MNPs were washed 2× with 1 mL MES buffer by using a magnetic separator and incubated with 5 mg of EDC in 250 µL of MES buffer for 10 min at RT and 750 rpm. After the activation, the MNPs were washed 2× with 1 mL MES buffer and resuspended in 125 µL of MES buffer. 0.17 nmol of capture sequence (PPRH-CC1, CC2 or CC3, and duplex-CC1, CC2, or CC3) were added and incubated for 2 h at 750 rpm. Then, the MNPs were again washed 3× with 1 mL of PBS and incubated in PBS containing 0.1% BSA for 2 h at 750 rpm. Finally, the solution was changed and the modified MNPs were resuspended in 1 mL PBS containing 0.05% sodium azide as preservative. Some 200 µL aliquots were prepared and stored in the fridge at 4 °C, until use.

The functionalized MNPs were used for sample pretreatment, which comprised the capture of the target sequence outside the biosensor device. An 83.3 µg/mL solution of MNPs modified with the capture sequence was prepared in TRIS buffer containing 1 mM EDTA and 1 M NaCl (hybridization buffer). To 300 µL of this suspension, 100 µL of a standard sample solution was added together with 100 µL of 83.3 nM HRP-conjugated reported sequence solution. Standard solutions containing increasing concentrations of the target DNA sequence (0, 0.01, 0.065, 0.41, and 2.56 nM) were tested. A first study was performed using the hybridization buffer to prepare the solutions. Thereafter, the evaluation was repeated but prepared the solutions of the target sequence in the Universal Transport Media from Biocomma Ltd., to simulate the real matrix of nasopharyngeal swab collected samples. In both studies, the mixture was incubated for 15 min at RT and 750 rpm. After this one-step incubation process, the MNPs were trapped with a magnet. The reaction solution was discarded and the MNPs were resuspended and concentrated in 100 µL PBS solution containing 0.05% Tween 20 (see Figure 7).

Once the sample was pretreated, some 7 µL of the MNP concentrated suspension was cast on the sample addition area of the fluidic channel in the electrochemical biosensor device and allowed to flow by capillary action. Solution took around 3 min to reach the area over the cartridge-inserted magnet where the MNPs were trapped. Then, a washing step was carried out by adding 7 µL citrate/acetate buffer solution and it was left to flow until all the solution disappeared from the sample addition area, taking around 6 min. Then, 7 µL of citrate/acetate buffer pH 5.5 containing 1.7 mM H_2_O_2_ and 2 mM ferrocene-methanol was added to the fluidic channel, and it was left to flow for 5 min. At this time, a chronoamperometric measurement at −0.15 V vs. Au CRE was carried out recording the current every 200 ms for 3 s. For more experimental details on the performance of the two-electrode electrochemical cell see reference [27].

The HRP label catalyzed the reduction of H_2_O_2_ using the ferrocene-methanol as electron donor, in situ generating its redox counterpart, that is ferrocenium methanol cation. This cation was reduced back to ferrocene-methanol at the electrode surface and the produced current signal was directly proportional to the concentration of the target sequence in the solution (see Figure 8). The current responses at 1.6 s were used as the analytical signal.

### 3.7. Determination of SARS-CoV-2 RNA in Clinically Relevant Samples

Samples and data from patients included in this study were provided by the Biobank of the Aragon Health System (PT20/00112), integrated with the Spanish National Biobanks Network and they were processed following standard operating procedures with the appropriate approval of the Ethics and Scientific Committees.

Three positive and three negative samples confirmed by PCR were tested by using the thermal lateral flow and the electrochemical biosensor systems. The six samples come from nasopharyngeal swabs that were collected from patients during the first year of the pandemic situation (August 2020). The UTM from Biocomma was used for the collection, which contains an inactivating agent for the positive samples. The samples were kept at −80 °C to preserve the viral RNA until the moment of measurement.

For the TLF system, 25 μL of sample either positive, negative, blank, or control, were tested by mixing the sample with 10 μg of nanoprisms functionalized with amino-modified first oligonucleotide PPRHs-CC1, 0.0638 nmol (26.6 μL) of the specific biotinylated second oligonucleotide and 23.4 μL of running buffer. 0.1 mL of this mixture was loaded directly into the strip cassette. The chromatography was performed for 15 min and after that, the strips were straightly irradiated by using a NIR laser (1064 nm, 1200 mW, 1 min). Positive samples provided a dark brown spot after laser irradiation in the back part of the strip, where the thermosensitive paper was included, and no signals were obtained for the negative ones.

For the electrochemical system, the samples were analyzed without any previous treatment, taking 100 µL and performing the measurement procedure described in the previous Section 3.6.

## 4. Conclusions

We have described the design and preparation of three triplex-forming oligonucleotides PPRH-CC1, PPRH-CC2, and PPRH-CC3 with high affinity for polypyrimidine sequences for the efficient capture and detection of SARS-CoV-2 genome. These oligonucleotides together with the appropriate reporter probes have been adapted to three biosensing devices including glass microarrays, thermal lateral flow devices, and electrochemical devices. In the thermal lateral flow device, the PPRH or duplex oligonucleotides are used for the functionalization of gold nanoprisms. Using glass microarrays, capture oligonucleotides were used for the functionalization of the glass surface. The trimolecular complex was detected after the hybridization of a mixture of analyte and the fluorescently (TAMRA or Cy3) labeled reporter probe. Limits of detection also depended on the UTM used and, in this analytical platform, the differences between duplex and PPRH capture probes are not so evident as in the previous biosensors, but it is possible to achieve values close to the ones observed with the previous devices.

In the thermal lateral flow system, the trimolecular complex is captured by streptavidin and developed by irradiation of an IR laser-generated heat that is collected in a thermal paper. Detection limits are between 0.005 and 0.01 nM. The best results were found with the PPRH-CC1 system. The PPRH and duplex-CC3 were not possible to be studied due to the aggregation of the nanoparticles. The PPRH-CC1 gave a limit of sensitivity one order of magnitude higher than duplex-CC1 (0.01 nM versus 0.1 nM).

The data from the electrochemical sensor gave similar results to the thermal lateral flow test. The trimolecular complex is detected using a peroxidase label that is directly attached to the reporter probe. Limits of detection slightly varied depending on the universal transport media (UTM) used but they were clearly better for PPRH oligonucleotides CC1 and CC3. The PPRH-CC2 system generated a lower signal, as well as duplex oligonucleotides, did.

The limit of detection of TENADA using synthetic oligonucleotides was around 0.01 nM (a few femtomoles) without the use of amplification steps. Detection of the presence of the RNA of the SARS-CoV-2 virus in nasopharyngeal samples agreed with the results obtained by PCR. The time required for the analysis is less than 1 h. The detection limit, the analysis time, and the requirement of simple instrumentation are strong arguments for further development. In addition, the triplex-assisted assay can be readily adapted for the detection of any pathogen with either DNA or RNA genomes as PPRHs are able to produce strand displacement when targeting duplex DNA [15,16,17,18] and it only requires the knowledge of the pathogen genome sequence.

The high affinity of PPRHs towards viral RNA can also be used to inhibit viral replication. For this reason, we are also studying the antiviral properties of CC1 and CC3 by infecting with SARS-CoV-2 virions VeroE6 cells previously transfected with the PPRHs. Results will be published when completed. Taking all these results together we demonstrate that non-canonical DNA structures and especially triplex nucleic acids can open new and interesting routes for improving biomedical applications of oligonucleotides such as the diagnosis of viral diseases.

## 5. Patents

A patent application has been filed (EP21382818.9, priority date 13.09.2021).

## Data Availability

Data will be made available from the authors on request.

## Supplementary Materials

The following supporting information can be downloaded at: https://www.mdpi.com/article/10.3390/ijms232315258/s1.
