# Supplementary material for: Detection of SARS-CoV-2 Virus by Triplex Enhanced Nucleic Acid Detection Assay (TENADA)
Source: Int J Mol Sci. 2022 Dec 3;23(23):15258. doi: 10.3390/ijms232315258 (PMC9740288; doi:10.3390/ijms232315258)
Supplement: Supplementary file 1 [file ijms-23-15258-s001.zip › ijms-2055625-supplementary.pdf]

SUPPLEMENTARY MATERIAL

Detection of SARS-CoV-2 virus by Triplex Enhanced Nucleic Acid Detection Assay (TENADA)

Anna Aviñó[1,2], Carlos Cuestas-Ayllón[2,3], Manuel Gutiérrez-Capitán[4], Luisa Vilaplana[1,2], Valeria Grazu[2,3], Véronique Noé[5,6], Eva Balada[1,2], Antonio Baldi[4], Alex J. Félix[5], Eva Aubets[5], Simonas Valiuska[5,6], Arnau Domínguez[1,2], Raimundo Gargallo[7], Ramon Eritja[1,2,*], M. Pilar Marco[1,2], César Fernández-Sánchez[2,4], Jesús Martínez de la Fuente[2,3], Carlos J. Ciudad[5,6]

1 Institute for Advanced Chemistry of Catalonia (IQAC), Consejo Superior de Investigaciones Científicas (CSIC), Barcelona, 08034, Spain; aaagma@cid.csic.es; lvhnqb@cid.csic.es; eva.balada@cid.csic.es; arnau.dominguez@iqac.csic.es; recgma@cid.csic.es; pilar.marco@iqac.csic.es;

2 Centro de Investigación Biomédica en Red de Bioingeniería, Biomateriales y Nanomedicina (CIBER-BBN), Instituto de Salud Carlos III, Madrid, Spain;

3 Instituto de Nanociencia y Materiales de Aragón (INMA), Consejo Superior de Investigaciones Científicas (CSIC), and University of Zaragoza, Zaragoza, Spain; carloscuestasayllon@gmail.com; vgrazu@unizar.es; jesusmartinezdelafuente@gmail.com

4 Instituto de Microelectrónica de Barcelona (IMB-CNM). Consejo Superior de Investigaciones Científicas (CSIC), Campus UAB, Cerdanyola, 08193, Spain; Manuel.Gutierrez@imb-cnm.csic.es; antoni.baldi@imb-cnm.csic.es; Cesar.Fernandez@imb-cnm.csic.es;

5 Department of Biochemistry and Physiology, School of Pharmacy and Food Sciences, University of Barcelona (UB), Barcelona, 08028, Spain; vnoe@ub.edu; ajife93@gmail.com; eva395@msn.com; simonasvaliuska@ub.edu; cciudad@ub.edu

6 Instituto de Nanociencia y Nanotecnología (IN2UB), University of Barcelona (UB), Barcelona, 08028 Spain;

7 Department of Chemical Engineering and Analytical Chemistry, University of Barcelona (UB), Barcelona, 08028, Spain; raimon_gargallo@ub.edu;

* Correspondence: recgma@cid.csic.es; Tel: +34-934006145; Fax: +34-932045904.

INDEX

Table S1. Results of TFO target sequences search in the SARS-CoV-2 genome.

Table S2. Sequences of the PPRH and reporter probes (RP) used in this study

Table S3. Sequences of the oligonucleotides prepared in this work.

Figure S1. Binding of CC2_DNA_target with NH_2-PPRH-CC2and NH_2-CC2duplex

Table S4. Dissociation constants of PPRH-CC1, CC2, CC3 and PPRH-CC1-control and duplex-CC1, CC2 and CC3.

Table S5. Limit of detection values (LoD, expressed in nanomolar, nM) corresponding to the CC pairs calibration curves obtained in a multiplex assay, testing different Universal transport media (UTMs). .

Table S1. Results of TFO target sequences search in the SARS-CoV-2 genome.

Sequences (5'-3')	Length	%GC	Orientation	Position
AGATGAGGATGAAGAAGAAGGTGA	24	41.7	Forward	3031
GAGCAGAAGGGTAGTAGAGAG	21	47.6	Reverse	17128
GTGATGAGGAACGAGAAGAGG	21	47.6	Reverse	28823
GAGGGAAGGACATAAGATGA	20	40.0	Reverse	2470

Table S2. Sequences of the PPRH and reporter probes (RP) used in this study.

Oligonucleotide	Sequence (5'-3')
**PPRH-CC1**	GAGCAGAAGGGTAGTAGAGAGTTTTGAGAGATGATGGGAAGACGAG
**PPRH-CC2**	GTGATGAGGAACGAGAAGAGGTTTTGGAGAAGAGCAAGGAGTAGTG
**PPRH-CC3**	GAGGGAAGGACATAAGATGATTTTAGTAGAATACAGGAAGGGAG
**CC1-RP**	GGCCAATAGCAAAATGACTC
**CC2-RP**	TGCGTAGAAGCCTTTTGGC
**CC3-RP**	CCCTTTCCACAAAAATCAAC

Table S3. Sequences of the oligonucleotides prepared in this work.

CC1 SYSTEM:
CC1PPRH-amino
5'-NH_2-TTTTTGAGCAGAAGGGTAGTAGAGAGTTTTGAGAGATGATGGGAAGACGAG-3'
CC1PPRH
5'-GAGCAGAAGGGTAGTAGAGAGTTTTGAGAGATGATGGGAAGACGAG -3'
CC1PPRH-Control (reverse Hoogsteen strand scrambled)
5'-GAGCAGAAGGGTAGTAGAGAGTTTTGGAGAGCAGGAATAGAGGAGT-3'
CC1duplex-amino
5'-NH_2-TTTTTGAGCAGAAGGGTAGTAGAGAG-3'
CC1biotineRP:
5'-GGCCAATAGCAAAATGACTC-BIOTINE-3'
CC1-TamraRP:
5'-GGCCAATAGCAAAATGACTC-Tamra-3'
CC1-Cy3RP:
5'-GGCCAATAGCAAAATGACTC-Cy3-3'
CC1-ThiolRP:
5'-GGCCAATAGCAAAATGACTC-Thiol-3'
CC1target:
5'-GAGTCATTTTGCTATTGGCCTAGCTCTCTACTACCCTTCTGCTC-3'
CC1DNAtarget-FAM
5'-FAM-GCTATTGGCCTAGCTCTCTACTACCCTTCTGCTCGCATAGTGTATAC-3'
CC1RNAtarget-FAM
5'-FAM-CUAGCUCUCUACUACCCUUCUGCUCGCAUA-3'

CC1DNAtargetLarge-FAM
5' FAM-GGTAAGAGTCATTTTGCTATTGGCCTAGCTCTCTACTACCCTTCTGCTCGCATA 3'

CC2 SYSTEM
CC2PPRH-amino:
5'-NH_2-TTTTTGTGATGAGGAACGAGAAGAGGTTTTGGAGAAGAGCAAGGAGTAGTG-3'
CC2PPRH:
5'-GTGATGAGGAACGAGAAGAGGTTTTGGAGAAGAGCAAGGAGTAGTG-3'
CC2duplex-amino:
5'-NH_2-TTTTTGTGATGAGGAACGAGAAGAGG-3'
CC2-biotineRP:
5'- TGCGTAGAAGCCTTTTGGC-biotine-3'
CC2-Cy3RP:
5'- TGCGTAGAAGCCTTTTGGC-Cy3-3'
CC2-ThiolRP:
5'- TGCGTAGAAGCCTTTTGGC-thiol-3'
CC2-biotineRPNew:
5'-CCTTCTGCGTAGAAGCCTTT -biotine-3'
CC2DNATarget:
5'-GCCAAAAGGCTTCTACGCAGAAGGGAGCAGAGGCGGCAGTCAAGCCTCTTCTCGTTCCTCATCAC-3'
CC2NewTarget:
5'-AAAGGCTTCTACGCAGAAGGGAGCAGAGGCGGCAGTCAAGCCTCTTCTCGTTCCTCATCAC-3'
CC2DNAtarget-FAM
FAM-5'- TCAAGCCTCTTCTCGTTCCTCATCACGTAGT-3'

CC3 SYSTEM
CC3PPRH-amino:
5'-NH_2-TTTTTGAGGGAAGGACATAAGATGATTTTAGTAGAATACAGGAAGGGAG-3'
CC3PPRH:
5'-GAGGGAAGGACATAAGATGATTTTAGTAGAATACAGGAAGGGAG-3'
CC3duplex-amino:
5'-NH_2-TTTTTGAGGGAAGGACATAAGATGA-3'
CC3-biotineRPnew1:
5'-CCCTTTCCACAAAAATCAAC-BIOTINE-3'
CC3-biotineRP:
5'- CCCTTTCCACAAAAATCAAC-biotine-3'
CC3-Cy3RP:
5'- CCCTTTCCACAAAAATCAAC-Cy3-3'
CC3-thiolRP:
5'- CCCTTTCCACAAAAATCAAC-thiol-3'
CC3DNAtarget:
5'-GTTGATTTTTGTGGAAAGGGCTATCATCTTATGTCCTTCCCTC-3'
CC3DNAtarget-FAM
FAM-5'-GTGGAAAGGGCTATCATCTTATGTCCTTCCCTCAGTCAGCACCTCAT-3'
CC3RNAtarget-FAM
FAM-5'-GGCUAUCAUCUUAUGUCCUUCCCUCAGUCA-3'

Figure S1. Binding of CC2-DNA-target with NH_2-PPRH-CC2and NH_2-CC2duplex

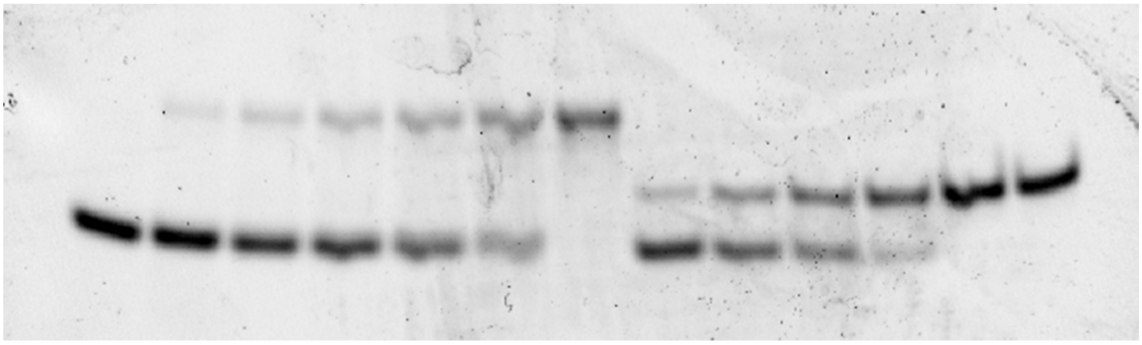

K_d PPRH = $6.04\cdot10^{-7}$ M K_d duplex = $7.47\cdot10^{-7}$ M

Table S4. Dissociation constants of PPRH-CC1, CC2, CC3 and PPRH-CC1-control and duplex-CC1, CC2 and CC3.

Oligonucleotide	K_d (10^{-7}), M
PPRH-CC1#	3.88
Duplex-CC1#	4.79
PPRH-CC2&	6.04
Duplex-CC2&	7.47
PPRH-CC3*	3.86
Duplex-CC3*	10.5
PPRH-CC1Control#	6.24

#Target oligonucleotide used: CC1DNAtargetLarge-FAM;
&Target oligonucleotide used: CC2DNAtarget-FAM;
*Target oligonucleotide used: CC3DNAtarget-FAM;

Table S5. Limit of detection values (LoD, expressed in nanomolar, nM) corresponding to the CC pairs calibration curves obtained in a multiplex assay. The table shows the data obtained when diluting the corresponding targets in different Universal Transport media (UTMs 1-3) compared with the values achieved when using hybridization buffer. In this case, the first oligonucleotides (PPRH and duplex format) were printed on the glass slide at a concentration of 125 nM. Subsequently, serial target dilutions were added (ranging from 500 nM to 0 with a dilution factor of 5). Finally, the labelled second oligonucleotides were added at an optimized concentration of 250 nM.

**Buffer/UTM**	**CC1**		**CC2**		**CC3**	
	**PPRH**	**Duplex**	**PPRH**	**Duplex**	**PPRH**	**Duplex**
**Hybridization buffer**	**1.01**	**0.64**	**3.16**	**4.67**	**1.56**	**3.49**
**UTM 1**	**0.47**	**0.61**	**2.53**	**2.60**	**2.58**	**3.76**
**UTM 2**	**0.01**	**0.06**	**2.03**	**1.23**	**0.72**	**0.33**
**UTM 3**	**0.03**	**0.02**	**0.89**	**0.71**	**1.50**	**1.17**
